# Global challenges of cancer imaging: perspective from different parts of the world: Asia

**DOI:** 10.1186/1470-7330-14-S1-O3

**Published:** 2014-10-09

**Authors:** Pek-Lan Khong

**Affiliations:** 1 Department of Diagnostic Radiology, Queen Mary Hospital, The University of Hong Kong

## 

Asia accounts for about 60% of the world's population and half the global burden of cancer [1]. Due to the diverse economic development across the Asian continent, countries have highly variable health services development and healthcare infrastructure. Thus, there is disparity in access to services, especially resource intensive cancer management. All countries in Asia, except Japan are defined by the UN as 'less developed regions' and this reflects the limited resources in general. This presentation aims to describe the demographic characteristics of cancer in Asia, and highlight the challenges and main advancements of cancer imaging across this region.

Lung cancer is the most common cancer in men (35.2/100,000 persons) and breast cancer the most common cancer in women (29.1/100,000 women) in Asia.

The incidence rate of breast cancer in Asia is about 1/3 compared to North America (NA) and Western Europe (WE), although it is on a steady rise. However, the five year prevalence rate is manifold lower indicating that in proportion, there are fewer cancer survivors in Asia. With the exceptions of Japan, South Korea and Taiwan, resources in most countries are inadequate for population-based organised screening mammography programmes. However, it is less clear if organised screening would be a cost-effective programme in Asia, due to the lower incidence rate as well as reduced sensitivity of mammography in Asian women with higher density breast tissue [2], and there is evidence that supplemental ultrasound screening increases the sensitivity for cancer detection in women with dense breasts [3].

Notably, in South East Asia (SEA) and East Asia (EA) compared to the rest of the world, the incidences of liver cancer, oesophageal cancer, stomach cancer and nasopharyngeal cancer are relatively high, although on the decline. Liver cancer is the second and third most common cancer in men in SEA and EA respectively, and fifth most common cancer in women. More than 70% of the new cases of liver cancer are from Asia, of which 50% are from China. Recent studies have advanced the use of gadolinium ethoxybenzyl dimeglumine (Gd-EOB-DTPA) enhanced MRI for its superior sensitivity for the detection of hepatocellular carcinoma, especially small tumors [4], and 11C-acetate PET imaging for detection of well-differentiated tumors [5]. Oesophageal cancer is the fifth most common cancer in EA men. Asia accounts to about 75% of new cases, specifically the 'asian oesophageal cancer belt' that extends from Turkey to Mongolia and Western/Northern China. The majority of these are squamous cell carcinoma in histology. Studies have found 18F-Fluorodeoxyglucose (FDG) PET to be useful in disease staging (particularly upstaging), and predictive of outcome of neo-adjuvant chemotherapy and disease survival [6]. Nasopharyngeal carcinoma is the sixth and fourteenth most common cancer in men across SEA and EA respectively. 80% of new cases are from this region, and are focussed around Southern China, Taiwan and SEA. Current research has evaluated the roles of diffusion MRI for tissue characterisation [7] and 18F-FDG PET for prognostication [8].

In recent years the cancer burden in Asia and its developing countries has surged, and is forecasted to continue to rise. Resources for the provision and development of diagnostic imaging which plays an essential role in early detection and management of cancer must be supported and strengthened across the region.
